# Southwestern Dental Society

**Published:** 1881-10

**Authors:** James W. White

**Affiliations:** Sec.; Carthage, Mo.


We have received the following:

CARTHAGE, MO., Aug. 24th, 1881.

INDEPENDENT PRACTITIONER,
Baltimore, Md.

The second annual meeting of the Southwestern Dental Society was held at Columbus, Kan., in the office of Dr. J. O. Houx, Aug. 9, 10 A. M., 1881. Dr. E. Hovey, of Springfield, Mo., President, in the chair. The following were elected officers for the ensuing year:

President—G. A. Keyes, Girard, Kan.
1st Vice-Pres't, J. O. Houx, Columbus, Kan.
2d " " S. J. Lindsey, Carthage, Mo.
Secretary—James W. White, Carthage, Mo.
Treasurer—C. F. Wright, Springfield, Mo.
Executive Committee—E. Hovey, C. F. Wright, E. W. LaViene.

JAMES W. WHITE, Sec.

